# Sustainable phyto-fabrication of silver nanoparticles using *Gmelina arborea* exhibit antimicrobial and biofilm inhibition activity

**DOI:** 10.1038/s41598-021-04025-w

**Published:** 2022-01-07

**Authors:** Smitha Chandrasekharan, Gandhimathi Chinnasamy, Somika Bhatnagar

**Affiliations:** Plant Transformation and Tissue Culture, Temasek Life Sciences Laboratory, 1 Research Link, National University of Singapore, Singapore, 117604 Singapore

**Keywords:** Nanobiotechnology, Nanoparticles

## Abstract

Increase in bacterial resistance to commonly used antibiotics is a major public health concern generating interest in novel antibacterial treatments. Aim of this scientific endeavor was to find an alternative efficient antibacterial agent from non-conventional plant source for human health applications. We used an eco-friendly approach for phyto-fabrication of silver nanoparticles (AgNPs) by utilizing logging residue from timber trees *Gmelina arborea* (GA). GC–MS analysis of leaves, barks, flowers, fruits, and roots was conducted to determine the bioactive compounds. Biosynthesis, morphological and structural characterization of GA-AgNPs were undertaken by UV–Vis spectroscopy, scanning electron microscopy (SEM), energy-dispersive spectroscopy (EDX), transmission electron microscopy (TEM), Fourier transform infrared spectroscopy (FTIR) and X-ray diffractometer (XRD). GA-AgNPs were evaluated for antibacterial, antibiofilm, antioxidant, wound healing properties and their toxicity studies were carried out. Results identified the presence of terpenoids, sterols, aliphatic alcohols, aldehydes, and flavonoids in leaves, making leaf extract the ideal choice for phyto-fabrication of silver nanoparticles. The synthesis of GA-AgNPs was confirmed by dark brown colored colloidal solution and spectral absorption peak at 420 nm. Spherical, uniformly dispersed, crystalline GA-AgNPs were 34–40 nm in diameter and stable in solutions at room temperature. Functional groups attributed to the presence of flavonoids, terpenoids, and phenols that acted as reducing and capping agents. Antibacterial potency was confirmed against pathogenic bacteria *Bacillus cereus*, *Escherichia coli*, *Pseudomonas aeruginosa*, and *Staphylococcus aureus* by disc diffusion assay, MIC and MBC assay, biofilm inhibition assay, electron-microscopy, cell staining and colony counting techniques. The results from zone of inhibition, number of ruptured cells and dead**-**cell**-**count analysis confirmed that GA-AgNPs were more effective than GA-extract and their bacteria inhibition activity level increased further when loaded on hydrogel as GA-AgNPs-PF127, making it a novel distinguishing feature. Antioxidant activity was confirmed by the free radical scavenging assays (DPPH and ABTS). Wound healing potential was confirmed by cell scratch assay in human dermal fibroblast cell lines. Cell-proliferation study in human chang liver cell lines and optical microscopic observations confirmed non-toxicity of GA-AgNPs at low doses. Our study concluded that biosynthesized GA-AgNPs had enhanced antibacterial, antibiofilm, antioxidant, and wound healing properties.

## Introduction

Unprecedented escalation in the frequency of antibiotic resistant bacteria has led to global health crisis with predictions of up to 10 million human deaths per year by 2050^1^. It is a challenge to constantly develop alternatives to classical antibiotics to slow down the widespread bacterial infections. Many pathogenic species are prevalent in communal areas and get easily transmitted through food, water, and soil, thus raising serious public health concerns. Virulent strains of *Escherichia coli* and *Bacillus cereus* are responsible for severe gastroenteritis in humans. *Pseudomonas aeruginosa* causes infections in immunocompromised and burn patients and *Staphylococcus aureus* infects skin and soft tissues. These bacteria have been reported to form resistant biofilms, colonize medical devices, implants, and wounds which often leads to morbidity and mortality^2^. There is dire need to substantiate conventional treatments with novel strategies to mitigate biofilm associated crisis. Novel technologies such as use of hydrogels as delivery vehicles can enhance penetration of antimicrobial agents into biofilm matrix for “on demand activation” and “smart release” of bioactive compounds. This multidisciplinary approach can leap forward the fight against antimicrobial resistance^3,4^.

Nanoparticles (NPs, 1–100 nm) offer diverse properties, such as size dependent crystalline structure, high surface area to volume ratio, stability, specificity and different electrical, magnetic, mechanical, optical, electronical characteristics than their bulk counterparts^5^. It has led to new applications in the electronic, water-purification, cosmetic, textile, food, feed, packaging, plant health and biomedical industry^6^. NPs are obtained from a wide range of material which can be broadly divided into organic material and inorganic material. Organic NPs such as lipid NPs (e.g. micells, liposomes) and polymeric NPs (e.g. dendrimers, ferritin, hydrogel) being biocompatible are used in drug-delivery systems for controlled release of therapeutic agents in precise dosage at specified sites. Inorganic material like carbon-based NPs (e.g. carbon nano tubes, quantum dots) are used as sensors and metal-based NPs (e.g. iron oxide, silver nanoparticles) are used for applications such as MRI contrast enhancement, tissue repair, immune assays, antimicrobial material etc.^7^. Among the different metals like iron, zinc, gold, silver, selenium, and nickel that are being explored for synthesis of NPs, silver has the advantage of chemical inertness, easy reduction, electrical conductivity, biosensor and wide implementation as an antimicrobial agent in form of silver nanoparticles (AgNPs)^8^. Physiochemical methods of NPs synthesis such as lithography, sputtering, laser ablation, photochemical reduction, aerosol technology, pyrolysis and ultraviolet irradiation require use of high temperature, toxic reactants, generate toxic by-products and often pose environmental challenges as well as safety control issues^9^. Green synthesis of NPs using bacteria, fungi, algae, and plants, has emerged as a fascinating alternative to these physical and chemical methods due to biocompatibility, self-reducing, capping and stabilizing abilities^10^. Compared with the use of single biomolecules like protein, peptide, enzyme, or DNA (which often degrade or get easily contaminated) or the microbes (which have sophisticated growth requirements and may exhibit pathogenicity), biosynthesis of AgNPs from plants is more advantageous as plants contain many bioactive molecules (polyphenols, terpenes, flavonoids, carboxylic acids, saponins, alcohols, and tannins), are economical to produce, and sustainable to use^11^. Valuable publications on green synthesis of AgNPs using plant extracts have been compiled in recent reviews^12,13^. AgNPs derived from trees like *Azadirachta indica*, *Bridelia retusa, Erythrina suberosa, Glochidion lanceolarium*, *Melia azedarach*, *Semecarpus anacardium*, and *Terminalia belarica* have been reported to have antimicrobial properties^14–18^.

*Gmelina arborea* (GA), commonly known as white teak, is a fast-growing perineal tree, economically valued for high-quality timber^19^. The trees are predominantly harvested for wood to manufacture plywood, packaging, furniture, paper-pulp, vehicle bodies and leave behind 25–45% of biomass (non-utilized branches, tops, stumps, barks, leaves, and roots) as logging residue^20^. Indian traditional medicine system uses GA leaf, bark, and root extracts for their antidiabetic, antimicrobial, anticancer, immunomodulatory, cardioprotective, analgesic, anthelmintic, rheumatoid, antipyretic, and blood detoxifying properties^21^.

**Figure 1:**
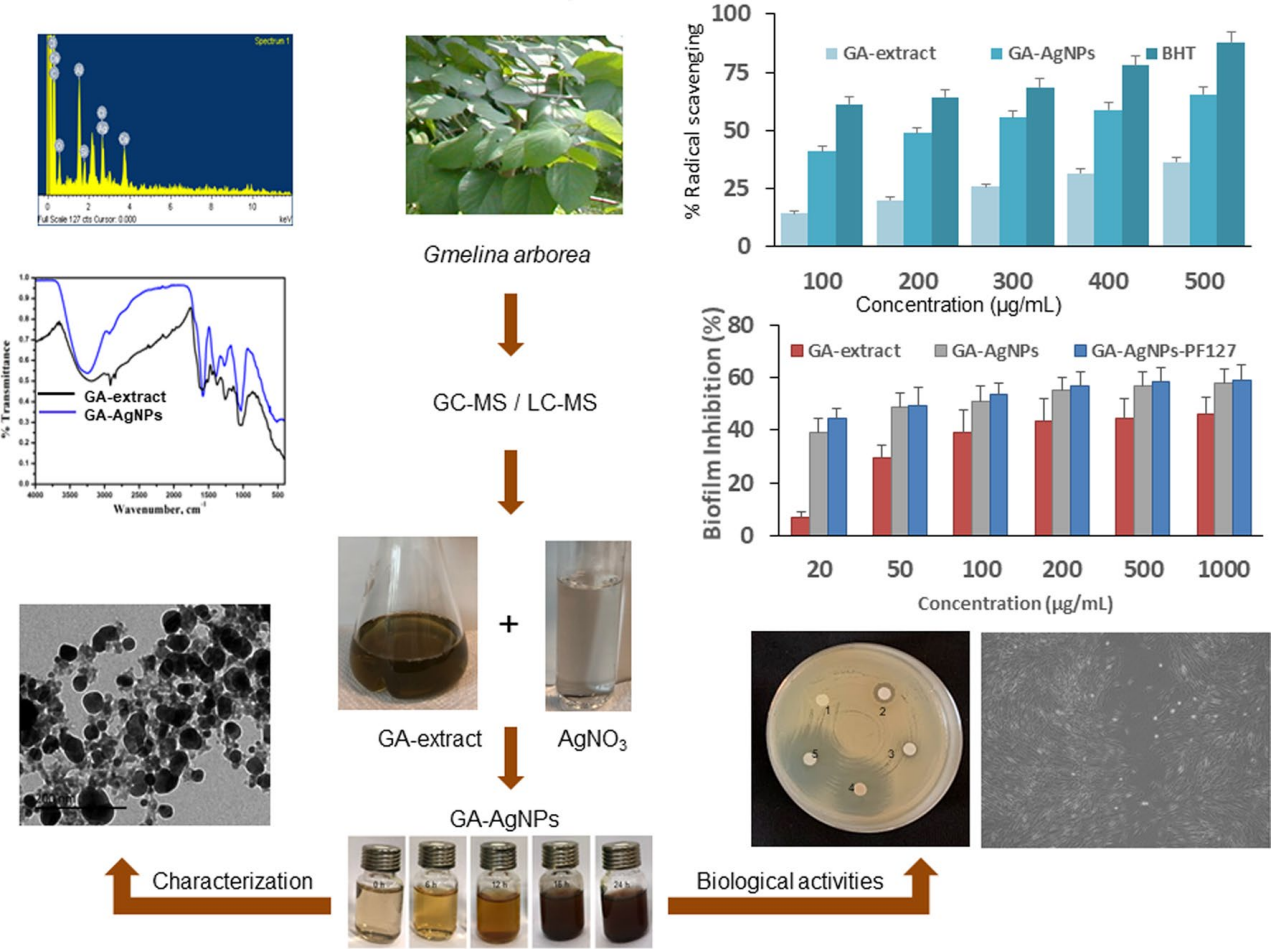
Phyto-fabrication of GA-AgNPs from leaf extract of *Gmelina arborea* and their characterization and biological activities.

The aim of present study is to bio-synthesize novel AgNPs using a sustainable source of raw material (logging residue from tree), with documented antimicrobial properties (GA tree), and explore a non-toxic, biocompatible, thermo-responsive hydrogel (PF127) as a delivery-vehicle of GA-AgNPs. Application of GA-AgNPs-PF127 with proven antimicrobial, antibiofilm, and antioxidant efficacy will inhibit bacterial growth, prevent oxidative damage, and accelerate healing of infected wounds. Towards this objective, in the present study, we extracted phytoconstituents from leaves, barks, flowers, fruits, and roots using polar and non-polar solvents and characterized them via GC–MS and LC–MS analysis. Based on these preliminary studies we further carried out biosynthesis of AgNPs using leaf extract. Reaction parameters of reactants concentration, pH, temperature, and incubation time were optimized for high yield and stability of GA-AgNPs. UV–Vis spectroscopy, SEM, EDX, TEM, FTIR, and XRD techniques were used to characterize the morphology, shape, size, and structure of the GA-AgNPs. GA-AgNPs were evaluated for antibacterial, antibiofilm, antioxidant, wound healing properties and their toxicity studies were also carried out (Fig. 1).

## Results

### Identification of bioactive compounds

Gas chromatography mass spectroscopy (GC–MS) analysis identified the presence of secondary metabolites as terpenes, sterols, aliphatic alcohols, esters, fatty acids, and aldehydes in different plant parts of GA (Table 1). Liquid chromatography mass spectroscopy (LC–MS) analysis of leaf extract confirmed the presence of important flavonoids such as luteolin, kaempferol, quercetin, isoquercitin, rutin, and astragalin. The results indicated that the leaves were rich source of phytochemicals.

**Table 1:** Major bioactive constituents present in *Gmelina arborea* as characterized by GC–MS analysis rendering the antimicrobial and antioxidant activity.

S. no.	Name	Formula	Flower	Fruit	Root	Bark	Leaf	Leaf
			Hexane					Ethyl acetate
1	Aromandendrene​​	C_15_H_24_					✓	
2	Acetoxytrimethylsilane	C_5_H_12_O_2_Si					✓	✓
3	Benzene, 1,2,4-trimethyl	C_9_H_12_					✓	✓
4	Benzene, 1,2-Dimethoxy-4-(1-propenyl)-	C_11_H_14_O_2_					✓	✓
5	2(4H)-Benzofuranone, 5,6,7,7a-tetrahydro-4,4,7a-trimethyl–	C_11_H_16_O_2_					✓	✓
6	Benzoic acid	C_7_H_6_O					✓	
7	α-Bergamotene	C_15_H_24_	✓					
8	Caryophyllene​​	C_15_H_24_					✓	
9	Caryophyllene oxide	C_15_H_24_O					✓	✓
10	Cedrol​​	C_15_H_26_O					✓	
11	Edulan I​​	C_13_H_20_O					✓	
12	γ-Elemene	C_15_H_24_					✓	
13	Epiglobulol​​	C_15_H_26O_					✓	
14	α-Farnesene	C_15_H_24_	✓			✓		
15	Farnesan	C_15_H_32_			✓			
16	n-Hexadecanoic acid	C_16_H_32_O_2_			✓			✓
17	3-Hexanol	C_6_H_14_O		✓	✓	✓		
18	α-Ionone	C_13_H_20_O				✓	✓	
19	Isobutyl acetate	C_6_H_12_O_2_			✓			✓
20	Limonene	C_10_H_16_			✓	✓		
21	Nerolidyl acetate​​	C_17_H_28_O_2_				✓	✓	
22	1-Octen-3-ol	C_8_H_16_O		✓	✓	✓	✓	
23	Oxirane, tetradecyl-	C_16_H_32_O		✓				
24	α-Selinene​​	C_15_H_24_					✓	
25	β-Sitosterol​​	C_29_H_50_O			✓	✓	✓	
26	3,7,11,15-Tetramethyl-2-hexadecen-1-ol	C_20_H_40_O			✓		✓	✓
27	Trans-2-hexenal	C_6_H_10_O	✓	✓			✓	
28	Trimethylsilyl laurate	C_15_H_32_O_2_Si	✓	✓			✓	✓

### Phyto-fabrication and optimization of GA-AgNPs

**Figure 2:**
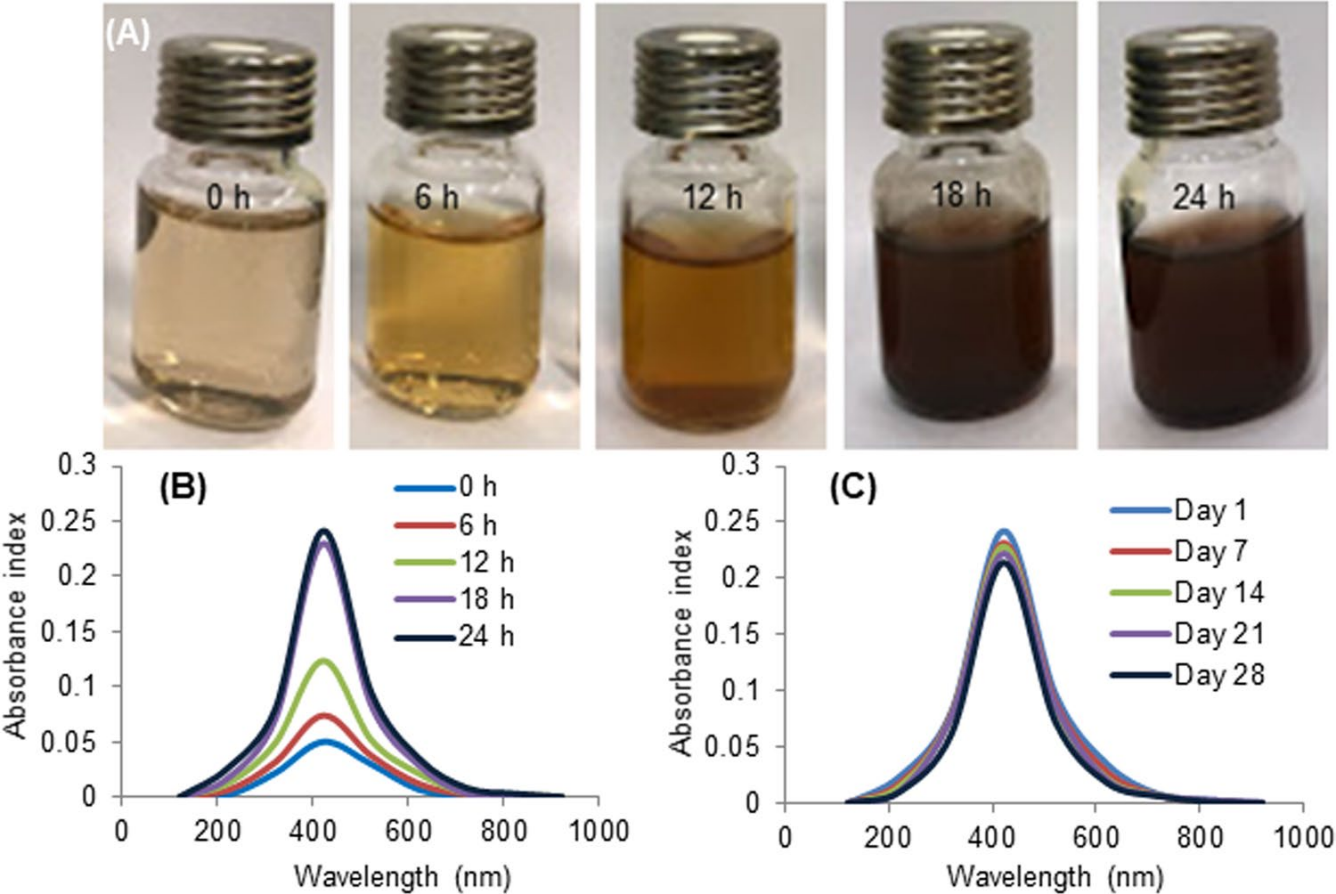
Phyto-fabrication of GA-AgNPs: (**A**) change in color during formation, (**B**) absorption spectra at various time intervals, and (**C**) on-shelf stability in UV–Vis spectroscopy analysis.

Addition of aqueous GA-extract to colourless silver nitrate (AgNO_3_) solution resulted in change in the colour of reaction mixture that intensified progressively from pale to dark brown (Fig. 2A). This gave qualitative confirmation about the formation of GA-AgNPs. Reduction of silver ion from Ag^+^ to Ag° was further confirmed by the absorbance maxima at 420 nm in UV–Vis spectroscopy. High yield of GA-AgNPs was obtained when GA-extract and AgNO_3_ were mixed in the ratio 1:9. The yield improved with increased concentration of AgNO_3_ up to 1 mM. The pH value of reaction mixture affected synthesis, with insignificant amount of GA-AgNPs formed at pH3, enhanced yield with small sized GA-AgNPs at pH7 and agglomerated particles at pH10. The amount of GA-AgNPs obtained when synthesized at low temperature (4 °C) was insignificant, reaction at the room temperature (RT, 25 °C) enhanced production of GA-AgNPs and the yield remain constant at higher temperature (37 °C). The increase in absorbance values confirmed the time dependent incremental yield of GA-AgNPs, with maximum yield attained at 18 h (Fig. 2B). This corresponded with change in colour of the reaction mixture. GA-AgNPs remained stable at RT during test and storage period of 28 days (Fig. 2C), as evident from the unchanged absorbance peak consistently appearing at 420 nm. Thus, results confirmed that the conditions optimized for production of high quality and quantity of GA-AgNPs were; 5 mL of GA-extract, 45 mL of 1 mM AgNO_3_, pH7, incubation at 25 °C, 200 rpm, dark for 18 h.

### Characterisation of GA-AgNPs

**Figure 3:**
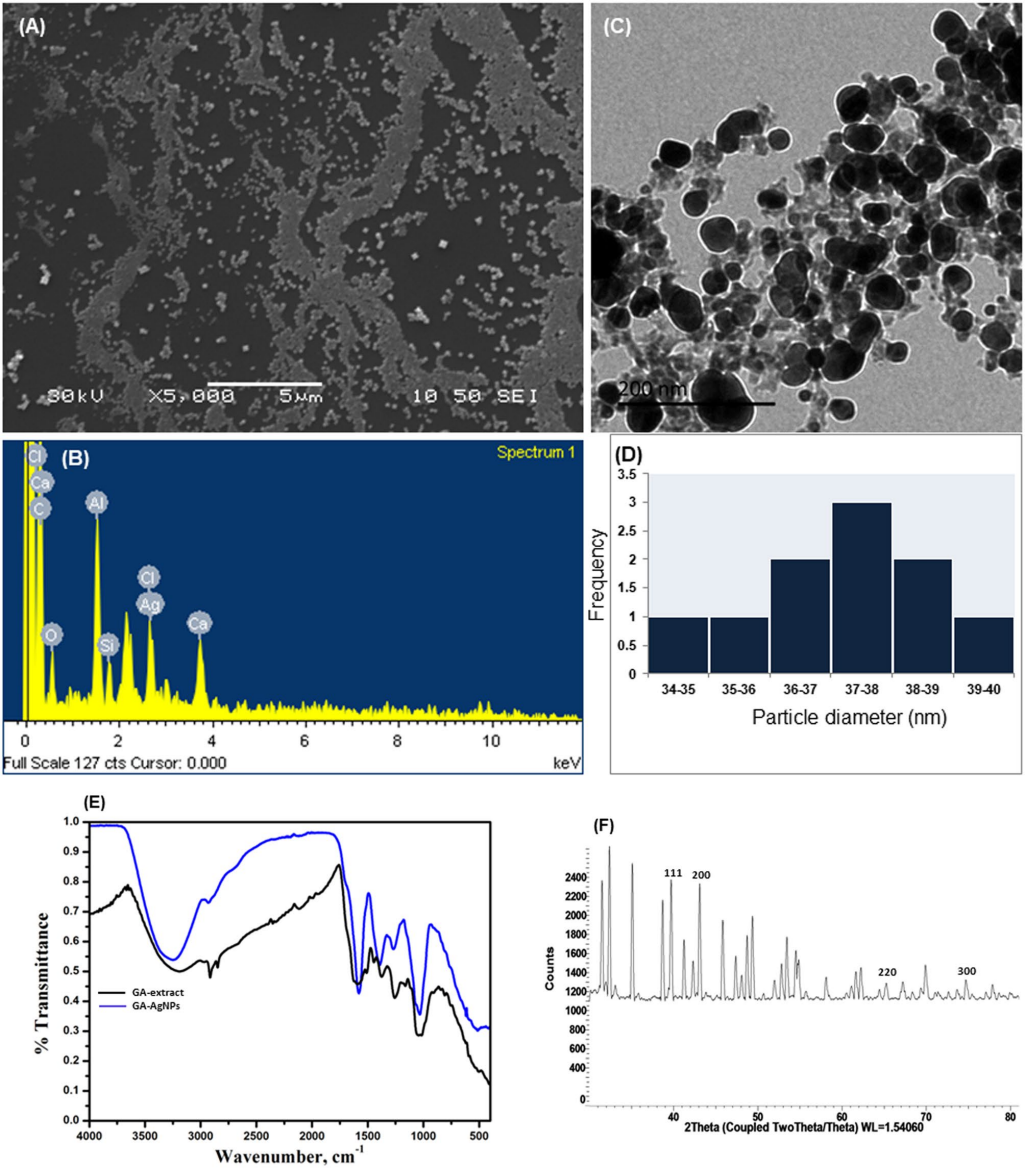
Characterization of phyto-fabricated GA-AgNPs: (**A**) SEM image, (**B**) EDX analysis, (**C**) TEM image, (**D**) particle size distribution, (**E**) FTIR spectral analysis, comparison with GA-extract and (**F**) X-ray diffraction analysis.

Scanning electron microscopy (SEM) image presented spherical shape of GA-AgNPs as the dominant form with a few cuboidal structures (Fig. 3A). Presence of elements was detected by energy-dispersive spectroscopy (EDX) analysis wherein the peaks corresponded to the atomic weight of each element (Fig. 3B). A strong signal around 3 keV confirmed the presence of Ag. The detection of other elements like oxygen (O), carbon (C), aluminium (Al), calcium (Ca), chlorine (Cl) and silicon (Si) indicated the presence of some extracellular moieties on the surface of GA-AgNPs. Transmission electron microscope (TEM) captured spherical and homogenously distributed GA-AgNPs (Fig. 3C). Average particle diameter observed was 36.38 ± 4.75 nm (Fig. 3D). In Fourier transform infrared spectroscopy (FTIR) of GA-extract and GA-AgNPs (Fig. 3E), common indicative peaks were 1037 cm^−1^ for –C=O– stretching vibration of alcoholic groups, 1254 cm^−1^ for –C–O– vibrations of hydroxyl flavonoids, 1455 cm^−1^ for bending vibration of –CH– for stretching vibration of aromatic –C=C– of CH_3_, CH_2_, flavonoids, and aromatic rings, 1644 cm^−1^ for –C=O– stretching vibrations of carbonyl/carboxylic groups, 2950 cm^−1^ for –CH– stretching vibration of methoxy/carboxylic groups and 3257 cm^−1^ for strong –OH–/–NH– stretching vibration of H-bond in alcoholic/phenolic or amino groups. Of special interest were those peaks which were present in GA-extract but absent in GA-AgNPs; 1185 cm^−1^ for –C–C– stretching vibrations of aromatic ring, 1480 cm^−1^ for –C=C– bending vibration of aromatic functional group, 1500 cm^−1^ for –C=O– stretching vibration of carboxylic group, and 2880 cm^−1^ for –CH– stretching vibrations of carbonyl group. X-ray diffractometer (XRD) results of the diffracted intensities measured at 2θ angles of 38.38°, 44.02°, 64.52°, and 76.66° were indexed to (111), (200), (220), and (311) lattice planes of crystalline face centered cubic structure of silver (Fig. 3F). The results confirmed characterization of biosynthesised GA-AgNPs.

### Antibacterial efficacy

**Figure 4:**
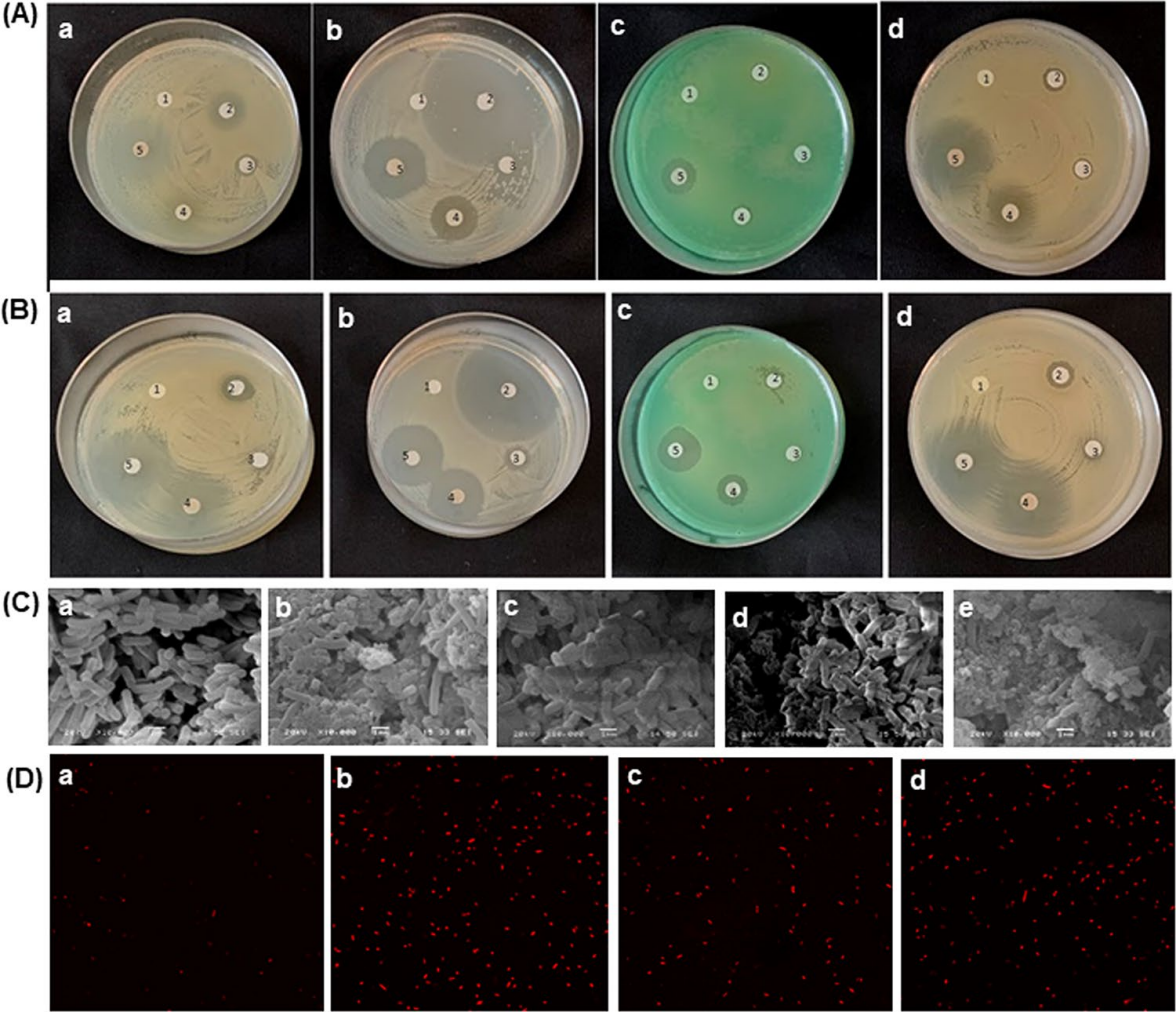
(**A**) Antimicrobial effect on (a) *B. cereus,* (b) *E. coli*, (c) *P. aeruginosa*, (d) *S. aureus,* in disc diffusion assay; control (1), Rifampicin (2), AgNO_3_ (3), GA-extract (4), GA-AgNPs (5), (**B**) antimicrobial effect on (a) *B. cereus,* (b) *E. coli*, (c) *P. aeruginosa*, (d) *S. aureus,* in disc diffusion assay; control (1), Rifampicin (2), PF127 hydrogel (3), GA-AgNPs (4), GA-AgNPs-PF127 (5), (**C**) SEM image of *E. coli* control (a), Rifampicin (b), AgNO_3,_ (c) GA-extract (d), and GA-AgNPs (e) and (**D**) Confocal microscopical dead cell image of *E. coli* control (a), Rifampicin (b), GA-AgNPs (c) GA-AgNPs-PF127 (d). Scale bar 10 µm.

In disc diffusion assay, zone of inhibition (ZOI) was observed as a circular marginated clear area on affluent bacterial culture plate that correspond to antibacterial potency of diffused samples from the paper-discs. Bacteria flourished well around the sterile distil water disc and AgNO_3_, while Rifampicin significantly inhibited bacterial growth. GA-extract exhibited some restriction in the growth of *B. cereus, E. coli, P. aeruginosa,* and *S. aureus* with ZOI measuring 10.0 ± 1.73 mm, 11.33 ± 0.57 mm, 6.6 ± 0.57 mm, and 11.66 ± 1.15 mm, respectively*.* Growth inhibition was more perceivable for treatment with GA-AgNPs at 15.6 ± 1.15 mm, 23.0 ± 1.73 mm, 13.3 ± 0.5 mm, and 18.3 ± 0.57 mm (Fig. 4A). Larger ZOI was recorded for GA-AgNPs-PF127 at 20.3 ± 1.15 mm, 30.0 ± 2.60 mm, 20.0 ± 1.0 mm, and 23.0 ± 2.0 mm respectively (Fig. 4B). The result indicated the magnitude of antibacterial activity was GA-extract ≤ GA-AgNPs ≤ GA-AgNPs-PF127.

### Bacteriostatic and bactericidal effect

Minimum inhibitory concentration (MIC) was the lowest concentration of a chemical that inhibits any visible growth of bacteria in Muller Hinton Broth (MHB) by microdilution method. When the MIC aliquots were seeded on Muller Hinton Agar (MHA) plates, the lowest concentration of GA-AgNPs required to produce no visible growth of that specific bacterium was defined as its minimum bactericidal concentration (MBC). These assays helped to evaluate the microbial potential of GA-AgNPs over a concentration range for a fixed duration of time under favorable bacterial growth conditions. The MIC and MBC values of GA-AgNPs ranged between 20 and 90 µg/mL and that of GA-AgNPs-PF127 at 10 µg/mL (Table 2). The results proved high antibacterial efficiency even at low concentration of GA-AgNPs and GA-AgNPs-PF127.

**Table 2:** Determination of diameter of zone of inhibition (ZOI), minimum inhibitory concentration (MIC) and minimum bactericidal concentration (MBC) of GA-extract, GA-AgNPs and GA-AgNPs-PF127 tested against four bacterial species.

Bacteria species	ZOI for GA-Extract (mm)	ZOI for GA-AgNPs (mm)	ZOI for GA-AgNPs-PF127 (mm)	MIC for GA-AgNPs (µg/mL)	MBC for GA-AgNPs (µg/mL)	MIC for GA-AgNPs-PF127 (µg/mL)	MBC for GA-AgNPs-PF127 (µg/mL)
*Bacillus cereus*	10.0 ± 1.73	15.6 ± 1.15	20.3 ± 1.15	20	20	10	10
*Escherichia coli*	11.33 ± 0.57	23.0 ± 1.73	30.0 ± 2.6	20	40	10	10
*Pseudomonas aeruginosa*	6.6 ± 0.57	13.3 ± 0.50	20.0 ± 1.0	90	90	10	10
*Staphylococcus aureus*	11.66 ± 1.15	18.3 ± 0.57	23.0 ± 2.0	90	90	10	10

### Antibacterial mechanistic effect

Mechanistic action was revealed by the morphological changes in bacterial cells. SEM image (Fig. 4C) presented regular rod-shaped *E. coli* cells of 3 µm × 1 µm size, with even surface, uniform, and intact cell structure in the control group. Treatment with GA-extract presented slightly distorted cells while the effect of GA-AgNPs was more drastic with cell wall disintegration and cytoplasmic leakage. Treatment with AgNO_3_ had negligible effect on cells and treatment with Rifampicin caused extensive damage. The confocal microscopy images in the live/dead cell staining assay confirmed that the red florescent dead cell population was higher in GA-AgNPs-PF127 and GA-AgNPs when compared with the control group (Fig. 4D). This indicated their high efficiency to kill bacteria.

### Biofilm inhibition

**Figure 5:**
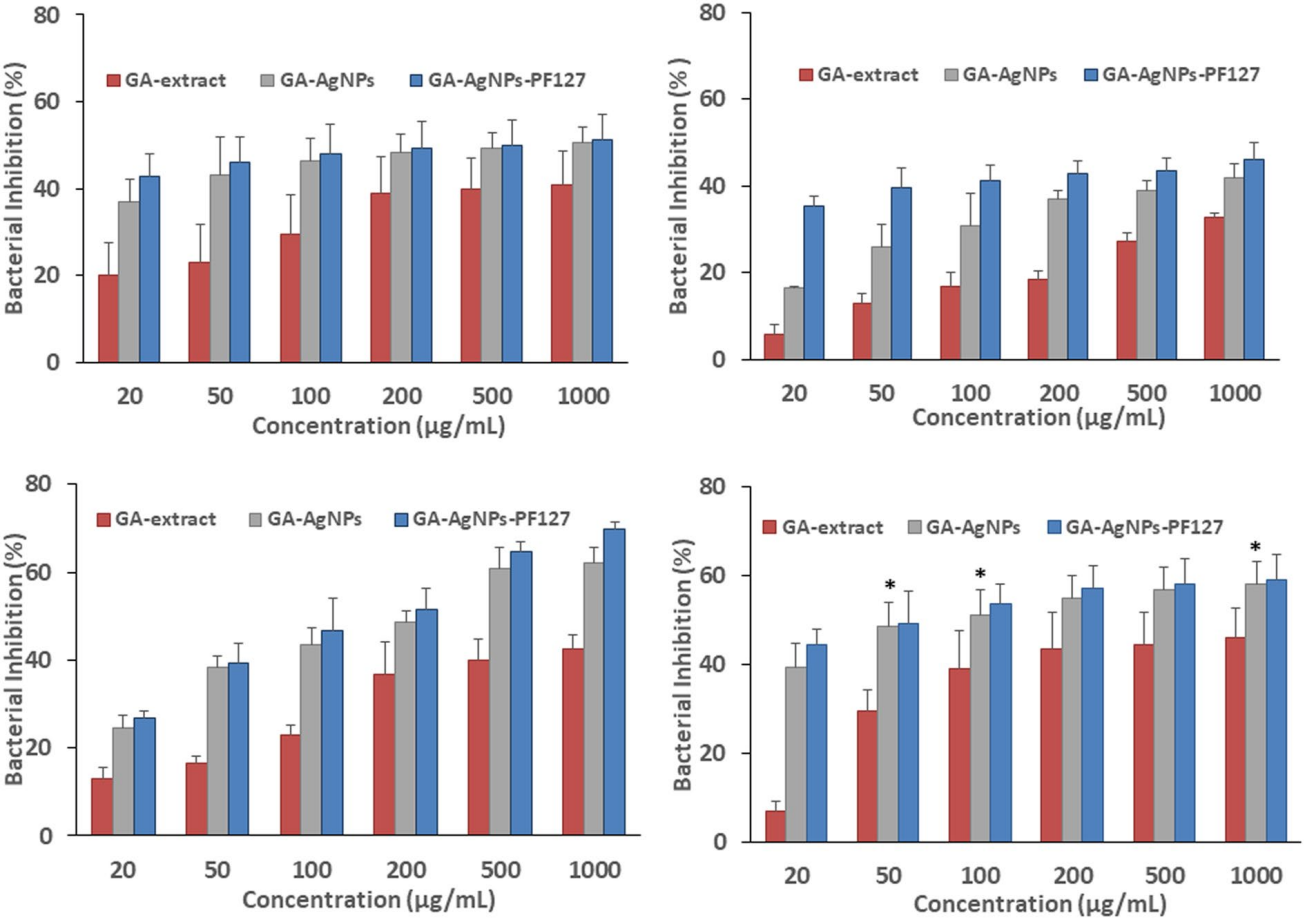
Biofilm inhibition effect of GA-extract, GA-AgNPs, and GA-AgNPs-PF127 on (**A**) *B. cereus*, (**B**) *E. coli*, (**C**) *P. aeruginosa*, and (**D**) *S. aureus.* Data represent mean value ± standard deviation. *p < 0.01.

In this study, effect of GA-extract, GA-AgNPs and GA-AgNPs-PF127 (20–1000 µg/mL) on biofilm inhibition was tested by crystal violet staining. The difference in effect was prominent at lower concentrations and reached a plateau with consistent inhibition at higher concentrations. Biofilm inhibition by GA-extract ranged between 6 and 46% and increased significantly to 24–62% for GA-AgNPs and 27–70% for GA-AgNPs-PF127; depending on the species of bacteria (Fig. 5A–D). Highest percentage of biofilm inhibition was attained by GA-AgNPs-PF127 with 51.12 ± 5.93 for *B. cereus*, 46.19 ± 1.23 for *E. coli*, 69.84 ± 1.66 for *P. aerunginosa* and 58.97 ± 5 for *S. aureus.*

### Bacterial cell viability

The antibacterial effect was further proved by cell viability assay, after exposure to GA-AgNPs, where log CFU/mL values did not show any growth suppression in sterile water and AgNO_3,_ while Rifampicin restricted cell growth. The result indicated that there was a > 90% reduction in bacterial cell viability (> 1 log reduction) after interaction with GA-AgNPs at 6.47 ± 0.16 log CFU/mL, which was higher than GA-extract at 6.84 ± 0.19 log CFU/mL.

Disc diffusion method, MIC and MBC values, SEM image, live/dead cell straining, cell viability assay and biofilm inhibition results together proved stronger bactericidal effect of GA-AgNPs.

### Antioxidant effect

**Figure 6:**
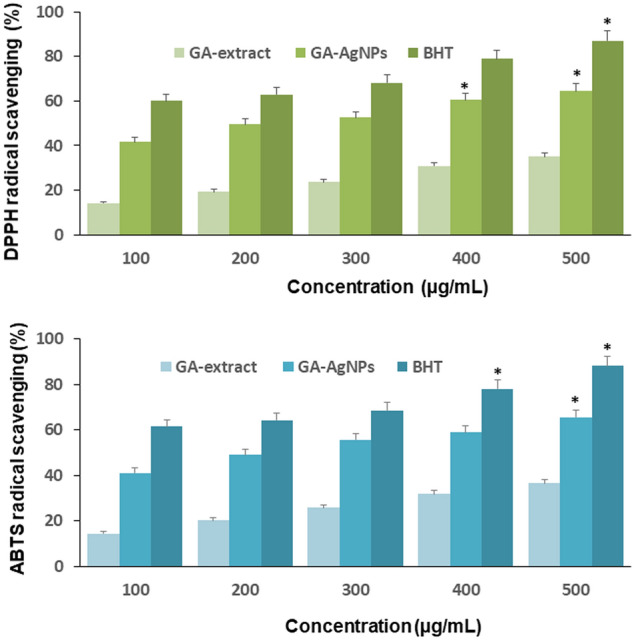
Antioxidant effect of GA-AgNPs: (**A**) DPPH radical scavenging and (**B**) ABTS radical scavenging. Data represent mean value ± standard deviation. *p < 0.001.

2,2-Diphenyl-1-picrylhdrazyl (DPPH) and 2, 2′-azino-bis (3-ethylbenzothiazoline-6-sulfonic acid) (ABTS) free radical scavenging assays confirmed a gradual increase in the antioxidant activity in a concentration-dependent manner. At 100 µg/mL, GA-extract showed 14.11% radical scavenging in DPPH assay and 14.4% in ABTS assay. These values increased to 35.06% and 36.46% respectively, at 500 µg/mL. 100 µg/mL of GA-AgNPs obtained 41.69% radical scavenging in DPPH and 41.04% in ABTS assay. Compared to GA-extract the percentage inhibition significantly (p ≤ 0.001) increased, 64.37% and 65.54% at 500 µg/mL GA-AgNPs in DPPH and ABTS assay. These results confirmed antioxidant potency of GA-AgNPs (Fig. 6A,B).

### Wound healing effect

**Figure 7:**
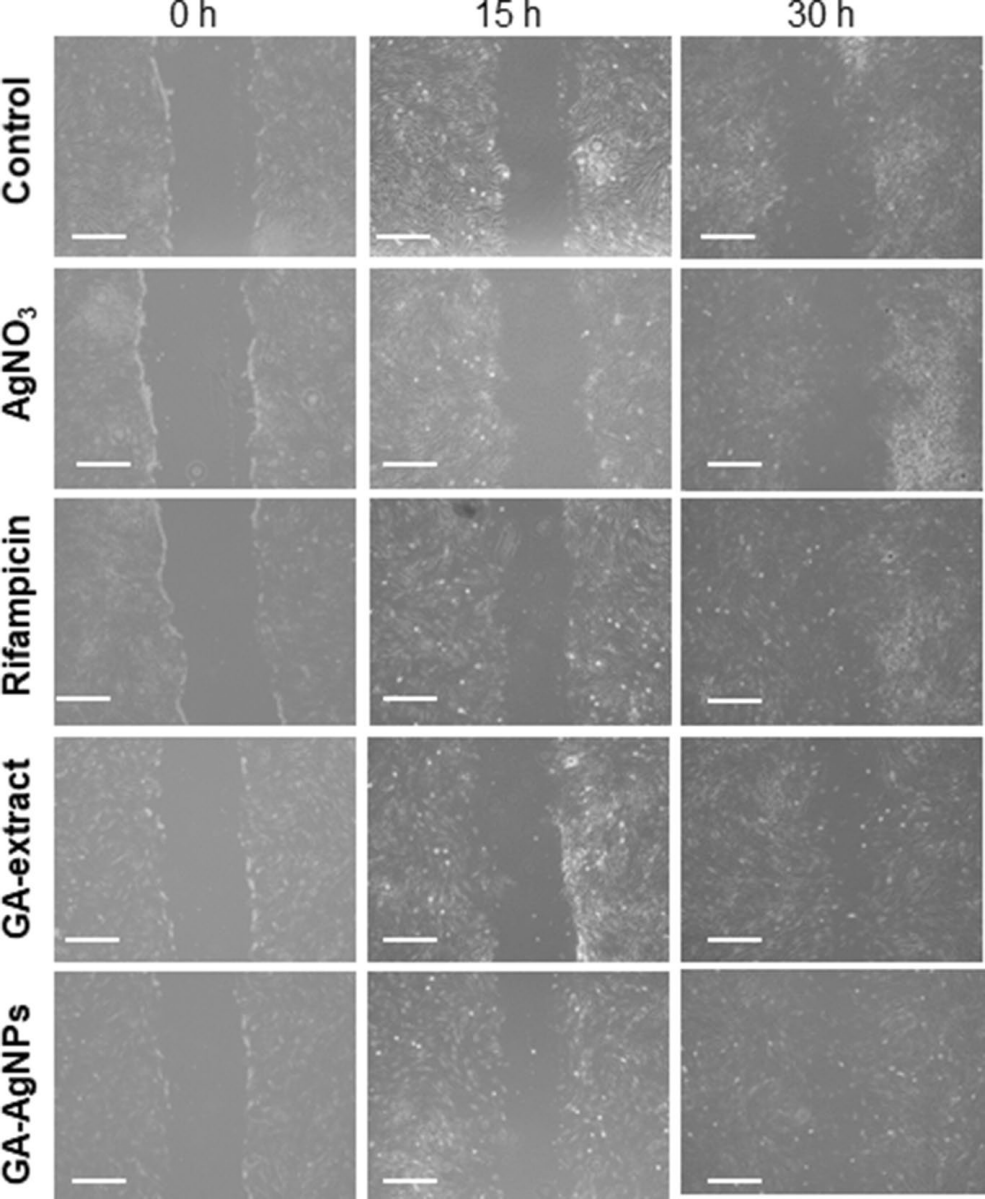
Wound healing effect of GA-AgNPs in cell scratch assay: at 0 h, 15 h and 30 h (×10 magnification) using human dermal fibroblast, (scale bar: 200 µm).

Cell migration plays an important role in wound healing. In the cell scratch assay conducted in human dermal fibroblast cell lines, the growth and migration of the cells in control and treated groups were observed after 15 h and 30 h of cell scratch (Fig. 7). In comparison with the control (34%), AgNO_3_ (41%) and GA-extract (58%) groups; the wound closure rate significantly (p < 0.01) increased in Rifampicin (68%) and GA-AgNPs (69%) treated groups at 30 h. This result implied potential of GA-AgNPs in wound healing process.

### Cytotoxic effect

**Figure 8:**
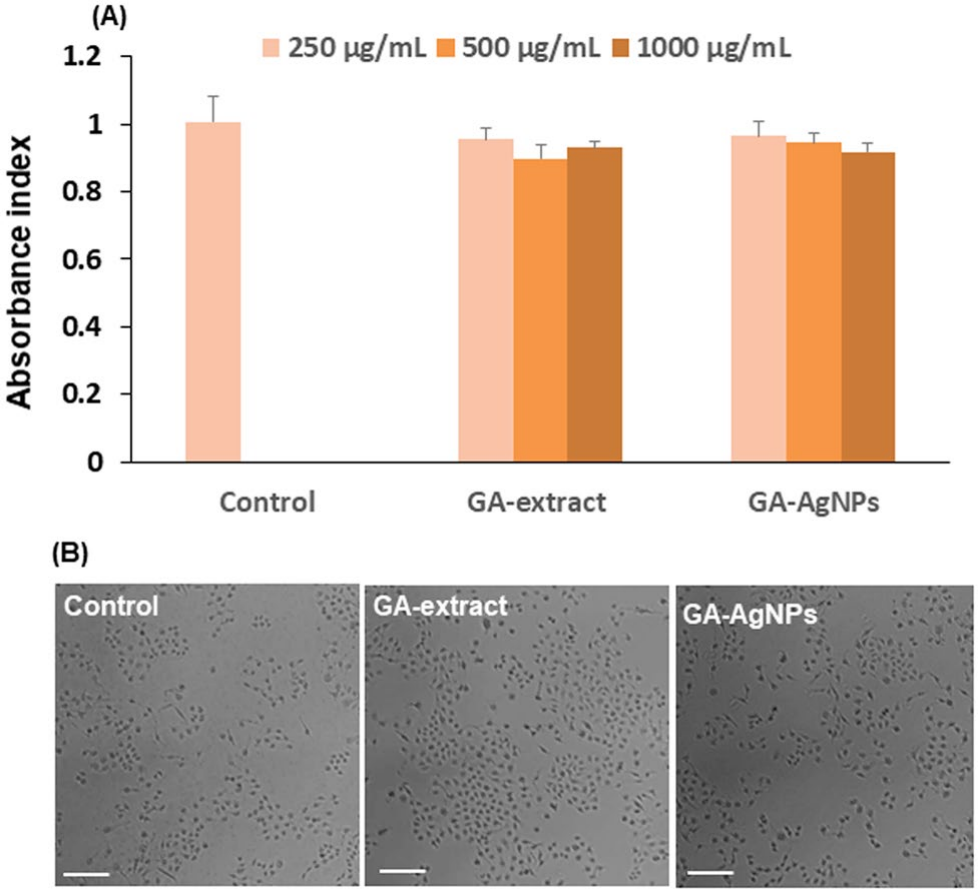
In vitro cytotoxic effect of GA-AgNPs: (**A**) MTS assay and (**B**) optical microscopic image on chang liver cell lines (scale bar 200 µm). Data represent mean value ± standard deviation. *p < 0.001.

The MTS assay {3, (4,5-dimethylthiazol-2-yl)-5-(3-carboxymethoxy phenyl)-2(4-sulfophenyl)-2H tetrazolium)} and optical microscopic analysis were conducted on human chang liver cell lines to study the cytotoxic effect of GA-AgNPs. Cell proliferation rate for GA-extract and GA-AgNPs was 95% and 96% in 250 µg/mL, 90% and 94% in 500 µg/mL, and 91% and 92% in 1000 µg/mL, respectively (Fig. 8A). No significant morphological change was observed in the cells treated with GA-extract and GA-AgNPs; they appeared like the control (Fig. 8B). These experiments confirmed low toxicity of GA-AgNPs.

## Discussion

Resistance to antibiotics and emergence of new pathogenic strains has led to profound economic losses due to diseases and illness. This warrants the search for new sources of antibacterial compounds and new ways of treatment. Screening of plants for compounds that can be converted into powerful drugs, is the modern scientific endeavor to reap the benefits of traditional systems of medicine. Plants contain secondary metabolites ideal for nanoparticle synthesis^22,23^. Different plant parts from diverse plant species have been explored for synthesis of NPs that are widely implemented for antimicrobial and biofilm inhibition purposes^15^. Towards harnessing the medicinal properties of Gmelina tree, our results have confirmed multiple applications of GA-AgNPs synthesized from its logging residue, the leaves.

Our GC–MS and LC–MS results confirmed an interesting repertoire of bioactive compounds especially in leaves of GA. Presence of flavonoids, lignans, iridoid glycosides, and sterols have been reported in Gmelina^24^. Major contributors to the antimicrobial property of medicinal plants are polyphenols, antioxidant activity is due to flavonoids, vitamins, polyphenols and anthocyanins; and wound healing activity is attributed to alkaloids, tannins, flavonoids, and terpenes^25^. Secondary metabolites are ubiquitously produced in plants that act as signalling molecules in plant–plant interaction and in plant defence response including biotic and abiotic stresses. They are also major contributors to the antimicrobial, antioxidant, anti-insecticidal properties of medicinal plants, have low toxicity in humans and are environmentally safe^26^.

Our environment friendly and biocompatible protocol for GA-AgNPs synthesis was carried out using universal solvent (water) for extraction and was conducted at room temperature. This makes the phyto-fabrication process more economical and feasible. The change in colour of transparent AgNO_3_ solution to brown upon addition of plant extract is considered as the visual confirmation of formation of AgNPs^9^. Upon excitation, the oscillatory motion of electrons in silver led to collisions, exhibiting surface plasma resonance (SPR) bands which are characteristic of AgNPs as detectable in UV–Vis spectroscopy. Distinct absorption peak of GA-AgNPs in our study was detected at 420 nm. Earlier studies have proved that the SPR peak for small size AgNPs (10 nm) was present around 392 nm while for larger AgNPs (100 nm) around 492 nm^27^. Concentration and ratio of reactants, pH, incubation time, temperature, and light are important factors that determine the quality and quantity of AgNPs and these parameters need optimization for desired yield of AgNPs^28^. In our studies, deviation from 1:9 ratio of GA-extract and AgNO_3_ resulted in lesser yield of GA-AgNPs due to unavailability of reactants. Though the formation of AgNPs has been reported over a wide range of pH from as low as pH3 to as high as pH10 subjected to plant source used; the optimum yield in the current study was obtained at pH7. The nature of the charged biomolecules to agglomerate varied with the pH range and thus affected metal ion reduction capabilities^29^. Room temperature was conducive for production of GA-AgNPs and increase in temperature did not produce any changes in the yield. Application of heat at high pH during AgNPs synthesis was reported to cause damage and denaturation of some active molecules, sugars and proteins rendering them inactive^29^. The incubation and storage of GA-AgNPs under dark conditions prevented photoactivation. The presence of light accelerated agglomeration of particles resulting in an unstable colloidal suspension^30^. During the formation of GA-AgNPs, initial SPR peaks were found to be broad and later narrowed down with the passage of time. After 18 h the peaks attained a constant shape, indicating the completion of reaction and exhaustion of available reactants. As the reaction progressed, amount of Ag^+^ that formed stable colloids with phytochemicals present in plant extract increased, this was evident from the sharpening of spectral peak indicating an optimal reaction time^31^.

AgNPs are morphologically and structurally characterized for particle size, shape, and nature by SEM, EDX and TEM analysis^32^. Our results indicated distinct, uniform, spherical nanoparticles of diameter 34–40 nm in SEM and TEM analysis. EDX analysis data showed strong signal for elemental silver around 3 keV and residue of other elements bound to surface of AgNPs; similar to earlier reports^32^. Polydispersity and morphology of AgNPs can be stabilized by optimum reaction conditions and metal salt reducing agents such as primary metabolites—proteins, organic acids, polysaccharides—and secondary metabolites—flavonoids and alkaloids^33^. Functional groups present in the biomolecules of synthesized GA-AgNPs attributed to the stabilization of the colloidal suspension by acting as capping and reducing agents are detected as multiple peaks on the spectrum of FTIR analysis. The stretching vibration between 1500 and 500 cm^−1^ represented functional groups such as –CN–, –CH–, –C–NH_2_, –C=O–, and –C=C–^34^. FTIR spectral analysis of *Osmium sanctum* extract and standard quercetin revealed distinct peaks for –C=C– and –NH–/–OH– at 1636 cm^−1^ and 3336 cm^−1^, and –C=O– and –NH–/–OH– at 1639 cm^−1^ and 3264 cm^−1^, respectively^35^. A similar peak observed in the spectrum of GA-extract inferred the presence of quercetin and its derivatives in Gmelina; this was also supported by our LC–MS results. In the comparative analysis spectra of GA-extract and GA-AgNPs, the missing peaks at 1185 cm^−1^, 1480 cm^−1^, and 1500 cm^−1^ indicated utilization of corresponding functional groups present in flavonoids in the formation of stable GA-AgNPs. In addition, absence of peak at 2880 cm^−1^ suggested that the electrostatic interaction between the carboxyl groups and free amines have resulted in protein precipitation that prevented agglomeration of the GA-AgNPs. Studies indicated that flavonoids act as reducing agents while terpenoids and phenols act as capping agents^36^. Hydrophilic functional groups of flavonoids bound to Ag^+^ yield colloid-stable GA-AgNPs in aqueous medium. Crystalline structure of GA-AgNPs was evident in XRD analysis and was similar to the other plant based AgNPs^37^.

Disc diffusion and microdilution assays were performed to examine the nature of aggregation of AgNPs in diffused and dispersed culture environments (MHA and MHB)^38^. The inhibitory effect of GA-AgNPs was observed in both Gram-positive (*B. cereus* and *S. aureus*)*,* and Gram-negative (*E. coli* and *P. aeruginosa*) bacteria. Our results indicated that the differential ZOI was correlated with the bioactivity of biomolecules that may have aggregated with Ag^+^ ion on the cell surface of GA-AgNPs in comparison with the GA-extract alone. Major contributors to this antimicrobial activity were β-sitosterol, epiglobulol, aromadendrene, caryophyllene, limonene, trans-2-hexenal, 1-octen-3-ol and 3-hexanol; based on similar findings in plants like *Azadiracta indica, Eucalyptus globulus, Malva parviflora,* and *Pinus eldarica*^14,39–41^. Recent reports have confirmed the bioactivity of AgNPs synthesised with kaemferol^42^, quercetin^43^, and rutin^44^ which are also found in GA-extract. Selective binding ability of these polyphenols reduces Ag^+^ ion to provide stability, greater exposed surface area and prevent agglomeration of the synthesised AgNPs^42–44^. Application of a combination of an antibiotic with an antibacterial-activity-enhancing agent (such as a plant extract), has been reported to be effective against antibiotic resistant bacteria in the new ‘combinational therapy’^45,46^. Hence, we plan to explore combination of GA-AgNPs with different antibiotics in our future experiments.

Qualitative analysis of GA-AgNPs by disc diffusion assay was followed by quantitative evaluation of its MIC and MBC. Lower MIC and MBC value confirm higher antibacterial potency of the synthesized GA-AgNPs and GA-AgNPs-PF127 against four bacterial species tested. These bacterial species have been reported to be sensitive to AgNPs at similar MIC and MBC values^47^. SEM images of Rifampicin and GA-AgNPs exposed bacterial cells depict significant detrimental effects of membrane lysis and cytoplasmic leakage as compared to control, AgNO_3,_ and GA-extract. SEM images revealed morphological alterations such as visible membrane pores, leakage of intracellular content, and cell lysis^47^. Live/dead cells staining assay showed presence of green/red fluorescent labelled cells as an effect of AgNPs on bacterial cells^47^. Our confocal microscopic image revealed a greater number of dead cell population in treatments with GA-AgNPs and GA-AgNPs-PF127 as compared to the control group. This proved that the bacterial growth inhibition effect of GA-AgNPs was enhanced when they were uniformly dispersed in the hydrogel. The ability of AgNPs to perturb the microbial cells is credited to its unique atomicity; smaller the size, higher the magnitude^48^. As established from characterization studies, the GA-AgNPs were in the size range of 34–40 nm with high surface area to volume ratio. This explains their stronger inhibitory effect than GA-extract. The synergy between nanoparticles and phytochemicals presents in the plants form target-site oriented phyto-nanoparticles that disrupt the membrane at the site of interaction. The acidic-basic affinity between Ag^+^ ions and sulfur containing proteins and phosphorous moieties of DNA molecules further enhance bactericidal capability^49^. Formation of reactive oxygen species (ROS) inhibited respiratory enzymes leading to cell death^50^. Broad spectrum inhibition of bacterial growth by GA-AgNPs established it as a pertinent advocate for antibacterial activity.

The mechanism of biofilm formation is a progressive process that begins with the reversible and irreversible attachment of microbial cells to a substrate, initiation of secretion of extracellular polymeric substances, followed by maturation or colonization and dispersal into individual cells^51^. The multi-layered aggregated bacterial cells alter the physiochemical properties of individual bacterial species. Under the compromised and altered environment of a biofilm, microbial cells alter its gene and protein expression to sequester exopolysaccharides to develop resistance to antibacterial agents. The slimy texture of exopolysaccharides in biofilm nourishes and protects microbial cells by improving its structural integrity and resistance. The altered cells are then released to adhere onto new surfaces thus improving its tolerance to antibacterial agents^52^. In our study, a gradient increase in biofilm inhibition was found to be proportional to concentration of GA-extract, GA-AgNPs and GA-AgNPs-PF127. However, the level of disruption was different in different species. This explains that the morphology of a biofilm alters with the bacterial species due to differences in mechanism of biofilm induction^53^. Reduction in CFU counts has been reported to signify reduction in bacterial cell viability following the AgNPs treatment^22^.

Over production of free radicals (which are unstable, highly reactive, capable of initiating chain reactions and causing damage to the cells) due to an imbalance in the oxidation–reduction equilibrium led to stress and cell death related diseases. It is thus vital to repress or deactivate these free radicals. Presence of thiols, polyphenols, ascorbic acid, and flavonoids in GA-extract that adhered on AgNO_3_ to form GA-AgNPs resulted in significant increase in the antioxidant efficiency when compared with the GA-extract in the DPPH and ABTS assays. Similarly, the free radical scavenging activity was reported to increase in AgNPs of *Teucrium polium* in a concentration dependent manner while no significant changes of antioxidant activity was observed in the chemically synthesized AgNPs^54^. Antioxidant activity of plant extracts were contributed by two major groups—polyphenols and carotenoids^55^ and within the polyphenols, tannins have more antioxidant potential than flavonoids^56^, this supports our similar findings in Gmelina.

Wound healing is a complex and dynamic process that involves four phases—homeostasis, inflammation, proliferation, and remodeling^57^. Proliferation and migration of cells such as keratinocytes and fibroblasts at the wound site leads to closing of wound^58^. Cell scratch assay is a suitable cost-effective method to validate cell migration, which is associated with the third phase of wound healing^59^. Chemically produced wound healing agents/drugs such as glucocorticoid steroids, non-steroidal anti-inflammatory drugs, chemotherapeutic drugs are invariably expensive, have side-effects, and are detrimental for the healthy tissue surrounding the wounds, thus plant extract derived AgNPs offer a feasible alternative^60,61^. Our in vitro cell scratch assay revealed enhanced cell migration in presence of GA-AgNPs. This was attributed to the presence of phytoconstituents. With activity levels like that of the antibiotic tested, GA-AgNPs qualify as a powerful candidate to be evaluated further for the wound healing efficacy in animal model in our future work.

Understanding of toxic effect is needed for the AgNPs that are intended for use in biomedical applications. Our results revealed that the cell proliferation and morphological changes in GA-AgNPs treated group were insignificant, thus confirming them to be safe for human usage in low doses. Rodents were found to be tolerant to various oral doses of Gmelina leaf extract with no apparent signs of toxicity including hematic, nephrotic, or hepatotoxicity^62^. AgNPs green-synthesized from *Oxal scandens* were found to be non-toxic while the chemically manufactured AgNPs manifested toxicity^63^. In biological extract based metallic nanoparticles, the metallic core is encapsulated with non-toxic molecules and thus these NPs become more biocompatible^9^.

In conclusion, our studies offer an environment friendly waste utilization opportunity by converting the residual material (leaves) into green nanoparticles using a cost-effective method at room temperature without using high temperature or toxic chemicals. Phyto-fabricated AgNPs were stable, monodispersed, crystalline, spherical, and uniformly sized (34–40 nm). The intrinsic bioactivity of leaf extract was manifested to higher levels in the form of GA-AgNPs. Persuasive antibacterial inhibition of both Gram-negative and Gram-positive bacteria made GA-AgNPs and GA-AgNPs-PF127 suitable candidates for further development of broad-spectrum antibacterial agent for healing of infected wounds. GA-AgNPs had potential antioxidant property and induced more cell migration, these will reduce formation of ROS at wound site. This study concludes that our phyto-fabrication method is simple, rapid, stable, repeatable, and amenable in scaling up for large-scale production of AgNPs in a sustainable way. These non-toxic phyto-fabricated GA-AgNPs have eminent antibacterial, biofilm, antioxidant, and wound healing properties.

## Materials and methods

### Material

Chemicals for this study were procured from Sigma-Aldrich, Singapore. Bacterial strains and cell lines were purchased from American Type Culture Collection, USA. *Bacillus cereus* ATCC 14579, *Staphylococcus aureus* ATCC 23235, *Escherichia coli* ATCC 25922, *Pseudomonas aeruginosa* ATCC 15442, human dermal fibroblast cells ATCC PCS-201-012, and human Chang liver cells ATCC CCL-13. Plant samples were collected in triplicate from GA trees from premises of Temasek LifeSciences Laboratory, Singapore.

### GC–MS and LC–MS analysis

GC–MS analysis was performed according to Chinnasamy et al*.* with slight modifications^14^ to identify the volatile phytoconstituents present in various parts of GA plant. Briefly, 1 g of thoroughly washed plant samples were lyophilized with liquid nitrogen and extracted in 1 mL of hexane or ethyl acetate with 10 mg/mL of camphor as internal standard. This mixture was incubated on a horizontal shaker at 50 rpm for 2 h. After centrifugation at 4500 rpm for 25 min, the organic layer was separated and dried with anhydrous sodium sulphate to remove any traces of moisture. After centrifugation, the filtrate was transferred to a glass vial and analyzed for the presence of bioactive compounds using GC–MS (7890 with 5975C Insert MSD with triple axis detector, Agilent Technologies, USA). 2 μL of sample was injected and separated at 50 °C for 1 min, temperature was increased at a rate of 8 °C per min to 300 °C and held for 5 min on a 30 m HP-5 MS column (Agilent Technologies, USA). MSD ChemStation Data Analysis (Agilent Technologies, USA) was used to identify compounds by comparing mass spectral data with NIST 2011 library.

To identify wider range of chemical compounds in leaves of GA, LC–MS analysis was performed^14^. 500 mg of leaf was extracted in 9 mL of methanol, centrifuged, and 5 µL of filtered extract was injected in LC–MS (Bruker Microtof II) separated using Chromolith Performance RP-18e 2.0 × 100 mm column and eluted with mobile phase of a mixture of distil water and acetonitrile (0.1% formic acid) at 0.2 mL/min.

### Preparation of extract for GA-AgNPs synthesis

Leaves were washed thoroughly to remove dirt and epiphytes, dried, coarsely grounded and sieved. 100 mL of distil water was added to 10 g of leaf-powder and incubated at 50 °C for 30 min with intermittent stirring for biomolecule extraction from leaf sample. Aqueous extract was cooled down to room temperature (RT, 25 °C), filtered through Whatman No. 1, labelled, and the filtrate was stored at 4 °C for further studies^17^.

### Phyto-fabrication of GA-AgNPs

GA-AgNPs were synthesized from the leaf extract^17^. Colour change and absorbance peak were monitored to determine phyto-fabrication. After completion of reaction, the nanoparticle suspension was centrifuged at 4500 rpm for 20 min to obtain a purified pellet of AgNPs and the supernatant was discarded. The pellet was washed thrice with distil water, air dried and stored in closed-dark vials at RT for further characterization and use. Optimization of essential parameters such as volume of GA-extract (1–10 mL), corresponding volume of 1 mM AgNO_3_ (49–40 mL), concentration of AgNO_3_ (0.5–2.0 mM), pH (3–10), duration of reaction (6–24 h) was performed at RT under dark. Aside, the on-shelf stability of GA-AgNPs was assessed periodically over a 28-day period. For all the experiments absorbance peak of GA-AgNPs was measured via UV-Spectrophotometer (UV1601, Shimadzu) with wavelength ranging from 100 to 900 nm.

### Characterization of GA-AgNPs

SEM (JEOL JEM-6360 OLV) was used to determine the morphology of GA-AgNPs after coating with gold (SCD 105 sputter coater). The elemental composition was investigated with EDX. TEM (JEOL JEM-1230) grids were prepared by adding a drop of sample in distil water dispersion on carbon-coated copper grid and drying it at RT. TEM was performed at an accelerating voltage of 40 kV to observe the space distribution and size of GA-AgNPs. FTIR (Thermo Fischer Scientific, Waltham, MA, USA) identified the functional groups responsible for bio reduction. The characteristic absorption spectra of GA-extract and GA-AgNPs at a frequency range of 4000–400 cm^−1^ was recorded. To acquire the spectra, the same equipment was operated in diffuse reflectance mode at 4 cm^−1^. To evaluate the crystalline nature of GA-AgNPs, XRD patterns was obtained with D8 X-ray diffractometer (Brucker Bioscience Corporation, USA) at a scan rate of 1 step/sec. The samples were exposed to Cu-Kα radiation over an angular range of 20°–80° (2θ).

### Preparation of GA-AgNPs-PF127

1 mg of GA-AgNPs were added to PF-127 hydrogel (30% w/v in cold PBS) and the mixture was incubated at 4 °C on 200 rpm for 18 h under dark to prepare GA-AgNPs-PF127.

### Disc diffusion assay

Antibacterial efficiency of GA-AgNPs was determined by disc diffusion assay, MIC and MBC assay, biofilm formation and disruption assay. The damage to bacterial cells was further assessed by CFU count, SEM and cell viability assay after exposure to AgNPs.

Antibacterial potential was tested against four bacterial species (*E. coli, S. aureus, B. cereus, P. aeruginosa*) by Kirby-Bauer Disc diffusion method^64^. Cultures grown on Muller-Hinton (MH) broth/agar at 28 °C, 250 rpm, dark were spread-plated on MHA plates at 1 × 10^6^ CFU/mL for 18 h at 37 °C. In one set of experiments sterile filter paper discs of 6 mm diameter were impregnated with Test Samples [namely, sterile distil water (control), Rifampicin (reference), AgNO_3_, GA-extract and GA-AgNPs at 1000 µg/mL] and transferred aseptically on previously inoculated bacterial plates. In another set of experiment, activity of GA-AgNPs was compared with that of PF127 hydrogel and GA-AgNPs-PF127. The plates were kept at RT for 1 h for diffusion of samples prior to incubation at 37 °C for 24 h. The susceptibility of bacteria to the inoculated discs were measured as the clear area that appeared around the discs and recorded as ZOI.

### Bacteriostatic and bactericidal micro-dilution assays

MIC and MBC values of GA-AgNPs and GA-AgNPs-PF127 were determined by micro-dilution broth assay as performed according to Chinnasamy et al*.*^14^ under the guidelines of Clinical Laboratory Standard Institute with slight modifications^65^. In brief, two-fold serial dilutions of GA-AgNPs suspension (stock solution of 50 mg/mL) were prepared in fresh MHB in a 96-well micro-titer plate. Bacterial inoculum at a growth density of 1 × 10^6^ CFU/mL was added in each of these well to make a final volume of 100 µL. Untreated bacteria in MHB were tested as growth control and GA-AgNPs suspension in MHB as aseptic control. 24 h incubation at 37 °C determined the endpoint concentration of antibacterial agent that can cause bactericidal effect. Optical density (OD) of micro-titer plates was measured at 570 nm in Tecan-Spark Microplate reader. 5 µL from each well was plated on MHA devoid of antibacterial agent overnight at 37 °C to determine MBC values. GA-AgNPs-PF127 was used in similar way.

### SEM imaging of bacteria

SEM imaging of bacteria was performed to examine the extent of morphological changes after treatment with GA-AgNPs. Bacteria cultures (1 × 10^6^ CFU/mL) were treated with test samples at 1000 µg/mL for 6 h then centrifuged at 3000 rpm for 30 min. The pellets were washed three times with phosphate-buffered saline (PBS) and then fixed with glutaraldehyde (2.5%, 30 min). The pellets were again washed with PBS, and dehydration process was carried out with increasing concentration of ethanol (30–100%), 15 min for each step. Fixed cells were dried overnight with amyl acetate and observed under SEM after sputter coating the sample with gold.

For bacterial cell staining, cells seeded at 5 × 10^5^ CFU per well in a 6-well plate were treated with sterile distil water (control), Rifampicin (reference), GA-AgNPs and GA-AgNPs-PF127 and grown at 37 °C on 250 rpm under dark for 4 h. Cells were washed thrice with PBS, incubated with propidium iodide (10 µg/mL) for 30 min, mounted in vetashield medium and visualize under Zeiss microscope (LSM 980)^22^.

### Biofilm formation and disruption assay

To evaluate the effect of GA-AgNPs on biofilm formation, assay was performed with reference to Singh et al.^22^ with slight modifications. Briefly, pre-inoculum of the sample bacteria was diluted to a concentration of 1–2 × 10^6^ CFU/mL with MHB and plated into 96-well plate. After 5 h of incubation at 37 °C, culture medium was removed and replaced with fresh medium with GA-AgNPs at different concentrations without disrupting the biofilm. The plate was incubated further at 37 °C for 18 h under dark. Planktonic cells were discarded by washing three times with saline buffer and biofilm was quantified by crystal violet assay (0.1% w/v). After 15 min the wells were washed off with sterile distil water to remove excess dye. The biofilm was allowed to air-dry prior to dissolving in 95% ethanol. The bounded crystal violet stain was agitated for 15 min and absorbance was measured at 570 nm in Tecan-Spark Microplate reader. Wells containing untreated cells were marked as control. The effect of GA-extract and GA-AgNPs-PF127 were tested in similar way. Percentage of biofilm inhibition was calculated with the following equation:

$$\% \text{ Biofilm inhibition }= [{\text{OD untreated cell}}-{\text{ OD treated cell }}]/{\text{ OD untreated cell}} \times 100$$

### Bacterial cell viability assay

To detect the viability of bacterial cells after exposure to AgNPs, the test was performed according to Ramalingam et al. with slight modifications^66^. The Test Samples were incubated separately in 6-well plates, with bacterial suspension at 1 × 10^6^ CFU/mL at 37 °C under dark for 18 h. This inoculum was serially diluted (10^–1^–10^–8^) and then plated on MHA at 37 °C for 24 h. The viable bacteria were counted using colony counter after 24 h of incubation.

### Free radical scavenging assays

2,2-Diphenyl-1-picrylhdrazyl (DPPH) and 2,2′-azino-bis (3-ethylbenzothiazoline-6-sulfonic acid) (ABTS) radical scavenging assays were used to investigate the in vitro antioxidant efficacy of AgNO_3_, GA-extract, and GA-AgNPs at concentrations between 100 and 500 µg/mL. DPPH assay was performed according to the standard protocol with slight modifications^56^. In short, 100 µL of 0.1 mM DPPH was added into 100 µL of a sample, incubated at RT for 30 min under dark and absorbance (A) was measured at 517 nm. ABTS assay was performed by standard procedure with slight modifications^67^. In brief, 10 mL of 7.4 mM ABTS was mixed with 10 mL of 2.45 mM ammonium persulfate and the reaction mixture was kept at RT for 16 h under dark. 100 µL of ABTS solution was then added into 100 µL of sample, incubated at RT for 20 min under dark, and absorbance was measured at 734 nm. In both experiments, Butylated hydroxytoluene (BHT) was used as a reference standard, and the reaction mixture without samples was used as control. The percentage inhibition was calculated using the following equation:

$$\% {\text{ Inhibition}} = ({\text{A}}_{{{\text{Control}}}} - {\text{A}}_{{{\text{Sample}}}} )/{\text{A}}_{{{\text{Control}}}} \times 100$$

### Cell scratch assay

In vitro wound healing activity was studied by cell scratch assay^17^. In brief, complete medium of DMEM was used to culture human dermal fibroblast cells (HDFa) on 6-well plate at a density of 5 × 10^5^ cells/well. Plate was incubated at 37 °C in a humidified environment containing 5% CO_2_ for 24 h. Culture medium was then removed, and the cells were washed thrice with PBS. Medium containing 1 mL of Test Samples at 1000 µg/mL were separately added into this plate. The cells were then scratched using a sterile yellow tip. Plate was observed under a Leica BM IRB microscope (10× magnification) for cell migration at two different time intervals: 15 h and 30 h. The following formula was implemented for calculating the wound closure:

$$\% {\text{Wound}}\;{\text{closure}} = 100 - ({\text{Empty}}\;{\text{area}}\;{\text{at}}\;30\;{\text{h}}/{\text{empty}}\;{\text{area}}\;{\text{at}}\;15\;{\text{h}}) \times 100.$$

### Cell cytotoxicity assay

Cytotoxic effect of GA-AgNPs on human chang liver cells (CCL-13) was tested using MTS assay {3, (4,5-dimethylthiazol-2-yl)-5-(3-carboxymethoxy phenyl)-2(4-sulfophenyl)-2H tetrazolium)} with slight modifications^68^. CCL-13 cells were seeded on Dulbecco’s Modified Eagle’s Medium (DMEM) supplemented with fetal bovine serum (10%) and penicillin (1%) in 24-well plates and grown at 37 °C in a humidified atmosphere containing 5% CO_2_ until 80% of cell confluence was achieved. Effect of GA-extract and GA-AgNPs (250–1000 µg/mL) on growth of CCL-13 cells was observed at a plating density of 5 × 10^3^ cells/well. The cultures were then incubated at 37 °C for 48 h. In an experiment, samples were aliquoted into a 96-well plate followed by the addition of MTS reagent (1:5 ratio of MTS in serum free media) in each well and incubated for 3 h at 37 °C. The optical density was measured at 490 nm by microplate reader (Infinite M200 Tecan). In another experiment, these treated cells were observed under the microscope to detect morphological changes. Untreated cells were used as a control in both the above experiments.

### Statistical analysis

All the experiments were conducted in triplicates. The experimental data was expressed as the mean value ± standard deviation (mean ± SD) and significant level was analyzed using Student’s t test. p ≤ 0.01/p ≤ 0.001 were considered statistically significant.

### Ethical approval

This article does not contain any studies with human participants or animals performed by any of the authors.

### Statement on plants

Authors declare that this research work does not contain any endangered plant species. The plant material was taken with permission from cultivated *Gmelina arborea* trees growing in premises of our research institute. Experimental research and field studies (either cultivated or wild), including the collection of plant material, comply with relevant institutional, national, and international guidelines and legislation.

## Data Availability

The datasets generated during and/or analyzed during the current study are available from the corresponding author on reasonable request.
